# Noradrenaline Acting on Alpha1 Adrenoceptor as well as by Chelating Iron Reduces Oxidative Burden on the Brain: Implications With Rapid Eye Movement Sleep

**DOI:** 10.3389/fnmol.2019.00007

**Published:** 2019-02-19

**Authors:** Abhishek Singh, Gitanjali Das, Manjeet Kaur, Birendra N. Mallick

**Affiliations:** School of Life Sciences, Jawaharlal Nehru University, New Delhi, India

**Keywords:** catecholamine, C6, cell death, lipid peroxidation, oxidative stress, synaptosome, vitamins C and E, Neuro2a

## Abstract

The noradrenaline (NA) level in the brain is reduced during rapid eye movement sleep (REMS). However, upon REMS deprivation (REMSD) its level is elevated, which induces apoptosis and the degeneration of neurons in the brain. In contrast, isolated studies have reported that NA possesses an anti-oxidant property, while REMSD reduces lipid peroxidation (LP) and reactive oxygen species (ROS). We argued that an optimum level of NA is likely to play a physiologically beneficial role. To resolve the contradiction and for a better understanding of the role of NA in the brain, we estimated LP and ROS levels in synaptosomes prepared from the brains of control and REMS deprived rats with or without *in vivo* treatment with either α1-adrenoceptor (AR) antagonist, prazosin (PRZ) or α2-AR agonist, clonidine (CLN). REMSD significantly reduced LP and ROS in synaptosomes; while the effect on LP was ameliorated by both PRZ and CLN; ROS was prevented by CLN only. Thereafter, we evaluated *in vitro* the effects of NA, vitamin E (Vit E), vitamin C (Vit C), and desferrioxamine (DFX, iron chelator) in modulating hydrogen peroxide (H_2_O_2_)-induced LP and ROS in rat brain synaptosomes, Neuro2a, and C6 cells. We observed that NA prevented ROS generation by chelating iron (inhibiting a *Fenton* reaction). Also, interestingly, a lower dose of NA protected the neurons and glia, while a higher dose damaged the neurons and glia. These *in vitro* and *in vivo* results are complementary and support our contention. Based on the findings, we propose that REMS maintains an optimum level of NA in the brain (an antioxidant compromised organ) to protect the latter from continuous oxidative onslaught.

## Introduction

The brain is metabolically highly active, resulting in the continuous production of a high amount of reactive oxygen species (ROS; Vallyathan and Shi, 1997; Seaver and Imlay, 2004), whose level is proportional to the neuronal activities (Dugan et al., 1995; Demaurex and Scorrano, 2009). On the other hand, as compared to other organs of the body, the brain possesses the least antioxidants (Friedman, 2011; Lalkovicova and Danielisova, 2016) while it is rich in iron (Keen et al., 1985; Wang et al., 2016b). These factors render the brain extremely vulnerable to oxidative insults (Friedman, 2011). The ROS induces membrane-lipid peroxidation (LP) and -rigidity, causing damage to DNA as well as proteins leading to neurodegeneration (Dröge and Schipper, 2007), acute, and chronic disorders (Valko et al., 2004; Galli et al., 2005). Therefore, we argued that the brain possibly possesses a unique mechanism associated with a unique instinct behavior that it controls to address and protect itself from oxidative onslaught.

The brain is rich in monoaminergic neurotransmitters including noradrenaline (NA), dopamine, serotonin and melatonin, which possess antioxidant property and may offer protection to the brain (Yen and Hsieh, 1997; Gulçin, 2008). Indeed, independent studies have shown that NA, by reducing LP and ROS generation, may protect the neurons (Troadec et al., 2001; Traver et al., 2005; Das et al., 2008). Rapid eye movement sleep (REMS), a unique behavior, normally maintains brain NA level (Mallick and Singh, 2011; Mallick et al., 2012). However, upon REMS deprivation (REMSD), the NA level is elevated in the brain (Porkka-Heiskanen et al., 1995; Mehta et al., 2017) and that by acting on adrenoceptor (AR) induces REMSD-associated neuronal damage (Majumdar and Mallick, 2005; Biswas et al., 2006; Jaiswal and Mallick, 2009; Ranjan et al., 2010; Somarajan et al., 2016). These isolated, independent, apparently contradictory findings raise an intriguing but fundamental question: what basic purpose does NA serve in the brain so that its level changes through sleep-waking-REMS states? Additional evidence was needed to reconcile why although NA exerts neuro-protection (Troadec et al., 2001; Traver et al., 2005; Patri et al., 2013), upon REMSD-elevated NA induces apoptosis and neurodegeneration (Biswas et al., 2006; Somarajan et al., 2016). To address these issues, we conducted *in vivo* and *in vitro* studies to understand the possible beneficial effect of NA and its possible mechanism of action.

Independent studies have shown that iron (Fe^2+^) induces ROS (Chung et al., 2005; Wang et al., 2016a) and its (iron) deficiency aggravates restless leg syndrome, which is associated with REMS-loss when the NA level rises (Porkka-Heiskanen et al., 1995; Lee et al., 2001; Allen and Earley, 2007; Patrick, 2007; Auerbach, 2014; Connor et al., 2017; Mehta et al., 2017). Therefore, we proposed that at the molecular level NA might modulate Fe^2+^, ROS, and membrane LP to alter their equilibrium (relative levels) and depending on the shift in equilibrium, it would provide either neuronal protection or damage. In this study, using *in vivo* (rat) and *in vitro* (synaptosomes and cell-lines) complementary models, we explored if the REMSD-associated elevated NA-induced neuronal damage was mediated by modulating Fe^2+^ and ROS levels. First, we evaluated the effects of REMSD and NA on ROS and LP levels in rat brain synaptosomes with particular reference to AR-subtype involved in the process. To explore the effectiveness of the anti-oxidative property of NA, we compared the effects of NA, vitamin (Vit) E, and Vit C alone, as well as upon hydrogen peroxide (H_2_O_2_)-induced ROS generation in synaptosomes prepared from rat brain. As a mechanism of action, we also observed that NA chelated Fe^2+^ and prevented ROS generation by inhibiting the *Fenton* reaction. Thereafter, in Neuro2a and C6 cells we confirmed that NA indeed reduced ROS generation in normal as well as in H_2_O_2_-treated cells and protected them from death. Our findings offer a long overdue molecular mechanism of NA-induced protection of the brain (neurons and glia) as well as the role of REMS in maintaining brain NA level in health and diseases.

## Materials and Methods

### Materials

Clonidine (CLN), 6-diamidino-2-phenylindole dihydrochloride (DAPI), 2′,7′-dichlorofluorescin diacetate (DCF-DA), desferrioxamine (DFX), Dulbecco modified Eagle’s medium (DMEM), ferrozine (FZ), H_2_O_2_, 3-(4,5-dimethylthiazol-2-yl)-2,5-diphenyltetrazolium bromide (MTT), NA, propidium iodide (PI), propranolol (PRP), prazosin (PRZ), thiobarbituric acid (TBA), Vit C, Vit E were procured from Sigma-Aldrich, St. Louis, MO, USA. Butylated hydroxyl-toluene (BHT), ferrous sulfate (FeSO_4_), ferric chloride (FeCl_3_) and iron/ferrous (Fe^2+^) chelator FZ, trichloracetic acid (TCA) were procured from Sisco Research Laboratories (SRL) India. All other chemicals were of analytical grade and were locally purchased.

### Animals

Fifty-five male inbred wistar rats (*Rattus norvegicus*; 250–280 g, which corresponds to approx. 6-month-old rats) obtained from the animal house facility of the Jawaharlal Nehru University were used in the study. The rats were randomly distributed into eight groups of five animals each for *in vivo* studies, while the rest were used to prepare synaptosomes for *in vitro* studies. They were maintained at a 12/12 h light/dark cycle and supplied with food and water *ad libitum*. The NIH guidelines National Research Council Guidelines (2011; Guide for the Care and Use of Laboratory Animals^1^) for the humane carrying out of research on animals have been followed and all the experimental protocols were approved by the Institutional Animal Ethics Committee of the Jawaharlal Nehru University. Every effort was made to use a minimum number of rats and to minimize pain and discomfort to the experimental rats while acquiring statistically acceptable data.

The standard flower-pot method was used for 4-days REMSD as reported earlier (Gulyani and Mallick, 1993; Das and Mallick, 2008; Somarajan et al., 2016). In brief, for REMSD the rats were maintained on a small (6.5 cm diameter) platform raised over surrounding water. To rule out non-specific effects, a group of rats were maintained in the same room on a larger (13 cm diameter) platform (LPC) raised over water under identical environmental conditions. Freely moving normal home cage grown rats (FMC) were used for collecting baseline data. Another control set, the recovery group (REC) included rats deprived of REMS for 4 days and then allowed 3 days in their normal home cages to recover from lost REMS. Thus, every set of experiment included one rat each of FMC, LPC, REMSD, and REC groups and five such sets were carried out. Additionally, to study the AR subtype involved for the action of NA, in another five sets of two rats each, one FMC and other REMSD rats were intraperitoneally injected with either PRZ (4 mg/kg) or CLN (0.1 mg/kg) 8 h before sacrifice to prevent the action or release of NA, respectively. The dosage and time of PRZ and CLN injections were based on our earlier report (Gulyani and Mallick, 1995).

### Synaptosome Preparation

At the end of the experiments, all the rats were decapitated after cervical dislocation (Mallick and Adya, 1999; Das et al., 2008; Baskey et al., 2009). The brains were quickly removed and homogenized in 10 volumes of ice-cold buffer containing 0.32 M sucrose and 12 mM Tris at pH 7.4 and synaptosomes prepared as described earlier. In brief, the brain homogenate was centrifuged for 5 min at 6,000 rpm (3,000 *g*) and the supernatant was further centrifuged at 12,000 rpm (11,000 *g*) for 20 min. The pellet was suspended in 1 ml of the homogenizing buffer, loaded onto 1.2 M and 0.8 M preformed sucrose density gradient and ultra-centrifuged in a swing-out rotor at 25,000 rpm (100,000 *g*) for 2 h. The synaptosomal fraction at the interface of 1.2 M and 0.8 M sucrose was re-suspended in the homogenizing buffer and processed for ROS and LP estimations. The synaptosomes were prepared within 2–3 h of brain extraction and under 4°C to minimize the loss of enzyme activities. The protein concentration in the synaptosomes was estimated by the Lowry method (Lowry et al., 1951).

Synaptosomes prepared from FMC rat brains were treated with H_2_O_2_ to induce ROS generation. To study the modulatory effect of NA, Vit E, and Vit C, the synaptosomes from FMC rat brains were first treated with one of these modulators alone or in combination before treating them with H_2_O_2_ and their respective effects on ROS levels estimated; five such sets were conducted. Further, to evaluate the role of iron in modulating the NA-, Vit E-, and Vit C-induced ROS levels, which in turn might influence LP, we estimated ROS levels after treatment with iron chelator, DFX. To minimize the photo-oxidation of NA, we always prepared a fresh NA solution for each experiment and protected it from light exposure until treatment as well as throughout the duration of the treatment.

### Estimation of Synaptosomal LP

Synaptosomal LP was estimated essentially following a (minor modification) previous report (Rehncrona et al., 1980). The synaptosomal sample (0.4 mg protein) mixed with 0.5 ml of 30% TCA and 0.5 ml of 52 mM TBA was incubated in a water bath shaker for 45 min at 80°C. The tubes were then ice-cooled and centrifuged (4,000 *g*) in a refrigerated centrifuge. The absorbance of the supernatant was read using a UV160 spectrophotometer (Shimadzu, Japan) against a blank at 532 nm and expressed as nmoles malonyldehyde (MDA) mg^−1^ protein.

### Estimation of Synaptosomal ROS

ROS was estimated (Lebel and Bondy, 1990) using a ROS-sensitive fluorescent probe DCF-DA that diffuses through the membrane. The DCF-DA gets into the synaptosomes and converted into non-fluorescent reduced form of DCF. The latter, in the presence of ROS, gets oxidized into a highly fluorescent form of DCF, which was estimated. First, the synaptosomes (0.05 mg protein) were incubated with DCF-DA at 37°C for 15 min in a water bath. At the end of the treatment, the reaction was terminated by adding 2 ml of ice-cold phosphate buffer (PB) at pH 7.4. Thereafter, the sample mixture was centrifuged at 12,500 *g* for 8 min and the pellet was resuspended in 2 ml of PB. The DCF fluorescence was estimated as a reflection of ROS generated using a microplate reader (Thermo Scientific Varioskan Flash, Finland) at 488 nm and 525 nm for the excitation and emission, respectively.

### Cell Culture

The Neuro2a (RRID:CVCL_0470) and C6 cells (RRID:CVCL_0194) were obtained from the National Centre for Cell Science cell repository (Pune, India). These cells were cultured in DMEM, supplemented with 10% FBS and 0.1% penicillin-streptomycin, and incubated in a humidified 95% air and 5% CO_2_ at 37°C. The cells were sub-cultured once they reached about 80% confluence.

### MTT Cell Viability Assay

Cell viability was estimated using an MTT assay, which is based on the ability of the viable cells to metabolize MTT into formazan (Mosmann, 1983). Neuro2a and C6 cells (2 × 10^4^ per well) were seeded in a 96 well plate. The effects of NA, H_2_O_2_, DFX, FeSO_4_, and Vit E, as compared to untreated controls, were evaluated for cell viability. After incubation for 24 h with the test substance, the medium from each well was aspirated. Subsequently, controls as well as treated cells were incubated with the medium containing 5 μg/ml MTT at 37°C for 3 h. Thereafter, the cells were washed twice with PB saline (PBS), the cellular formazan crystals were solubilized with DMSO and absorbance recorded in a microplate reader (BioTek, Winooski, VT, USA) at 570 nm with background subtraction at 630 nm. The cell viability in the experimental set has been expressed as a percentage (mean ± SEM) change relative to the corresponding untreated control set (taken as 100%).

### Estimation of Intracellular ROS in Neuro2a and C6 Cells

The Neuro2a and C6 cells were grown in separate petri dishes in a CO_2_ incubator in a normal and iron-rich (10 μM FeSO_4_ added) medium and were treated with either or in various combinations of NA, Vit E, H_2_O_2_ and DFX for 2 h. The treated cells were washed and incubated in a medium containing 10 μM DCF-DA for 20 min. At the end, the cells were washed with an ice cold PB at pH 7.4. DCF-DA gets into the cells and is converted into a non-fluorescent form DCFH, which in the presence of ROS gets oxidized into highly fluorescent DCF. The intensities of DCF fluorescence in control and treatment groups were estimated using a spectrofluorometer (Agilent Technologies, Palo Alto, CA, USA) as a reflection of the quantity of ROS generated by the cells.

### Estimation of LP in Neuro2a and C6 Cells

Neuro2a and C6 cells (~2 × 10^7^ cells) were plated in separate 100 mm dishes. Cells were treated either with NA, Vit E, PRZ, PRP, H_2_O_2_ alone or in various combinations. At the end of 12 h treatment, the cells were washed and pelleted down and 30 μl of the BHT solution was added to it to stop further oxidation. Cell suspension was made in 0.5 ml of 30% TCA. Thereafter, 0.5 ml of 52 mM TBA was added and the mixture was incubated in a water bath shaker for 45 min at 80°C. The mixture was then ice-cooled and centrifuged (at 4,000 *g*) at 4°C for 15 min. The absorbance of the supernatant was read against a blank at 532 nm using a microplate reader (BioTek, Winooski, VT, USA) and expressed as nmoles MDA mg^−1^ protein.

### Iron (Fe^2+^) Chelation Assay

Fe^2+^ chelation studies were performed using the method reported earlier (Dinis et al., 1994). In a separate experiment, 200 μL of 10 μM to 1 mM of NA was mixed with 20 μl of 5–50 μM of either FeSO_4_ or FeCl_3_ and allowed to stand at room temperature for 30 min. At the end, 100 μl of 1 mM FZ was added to it, shaken vigorously and left for 10 min. The absorbance of the Fe^2+^-FZ complex in the solution was then estimated in a microplate reader (Biotek, Winooski, VT, USA) at 562 nm. All the tests were run in triplicate and their means taken for analyses.

### Fluorescence Microscopy for Intracellular DCF Fluorescence

Neuro2a and C6 cells (2 × 10^5^ cells) were seeded in separate petri-dishes having coated coverslips. Twelve hours of incubation were allowed for the cells to adhere on the coverslips and they were treated for 2 h either with NA or H_2_O_2_ alone or in combination. The treated and untreated cells were then incubated with a 10 μM DCF-DA loading buffer for 30 min, washed and mounted in Mowiol. The cells were manually outlined and the fluorescence per cell was estimated using a fluorescence microscope (Nikon Eclipse 400). One-hundred to one-hundred and twenty cells from 3–4 fields of view from each coverslip per treatment were estimated in duplicate and five such sets of experiments were carried out. The mean background intensity was subtracted to obtain intracellular fluorescence per cell. Mean fluorescence intensities (a.u.) per cell between different treated groups were statistically compared against that of the untreated group.

### Estimation (Qualitative) of Dead/Live Cells by PI Staining

Neuro2a and C6 (2 × 10^5^ cells) were grown on separate coverslips for 12 h before treating for 24 h with either NA or H_2_O_2_ alone or both in combination. The cells were then incubated with 500 nM PI (stains nuclei of dead cells) for 30 min prior to fixing with 4% paraformaldehyde followed by staining with 30 nM DAPI. The nuclei were stained with DAPI to rule out non-specific PI staining. The images were captured using the Olympus BX51 microscope equipped with a CS9000 camera (MBF bioscience, Williston, VT, USA). The cells stained with both DAPI (blue) and PI (red) were considered to represent dead cells, which needed quantification. However, the PI staining was done on coverslip grown cells, which are prone to and get detached when dead and hence, counting the remaining cells would compromise accuracy in the result. Therefore, we consciously avoided quantifying the PI stained dead cells and instead performed an additional experiment with trypan-blue staining as described below.

### Quantification of Dead Cells by Trypan Blue Assay

Neuro2a and C6 (2 × 10^5^ cells) were separately seeded in 12-well culture plates for 12 h and the cells were treated with either NA or H_2_O_2_ alone or both together for 24 h. The cells were then dissociated, centrifuged and re-suspended in 1 ml PBS. Ten microliter of such cell suspension was then mixed with an equal volume of 0.4% trypan-blue stain for 3–4 min; the trypan-blue is taken up by the dead cells. Thereafter, the stained (dead) as well as unstained (live) cells (i.e., total number of cells) were counted using a hemeocytometer. The percentage of stained cells to total cells (stained plus unstained) was used as an estimate of dead cells and compared statistically between the treated and untreated cells.

### Statistical Analysis

The data has been presented as mean ± SEM. A statistical analysis was carried out using SigmaStat 3.0 (RRID:SCR_010285, Jandel Scientific, San Rafael, CA, USA). To compare data from different groups within an experimental set-up, all pairwise multiple comparison test using one way analysis of variance (ANOVA) was applied; it compared values among groups as well as between control and treatments. A Tukey test was applied for evaluating the significance level; at least *p* < 0.05 was considered statistically significant. The results of statistical tests have been presented as *F*_(x,y)_ followed by *p*-values, where *F* is the ratio of variance between samples or groups and the variance within samples or groups, while *x* and *y* are degrees of freedom between the groups and within the groups, respectively.

## Results

### Studies in Rat Brain Synaptosomes With or Without Treatment *in vivo*

#### LP and ROS Were Reduced in Synaptosomes Prepared From REMS-Deprived Rats

**Figure 1:**
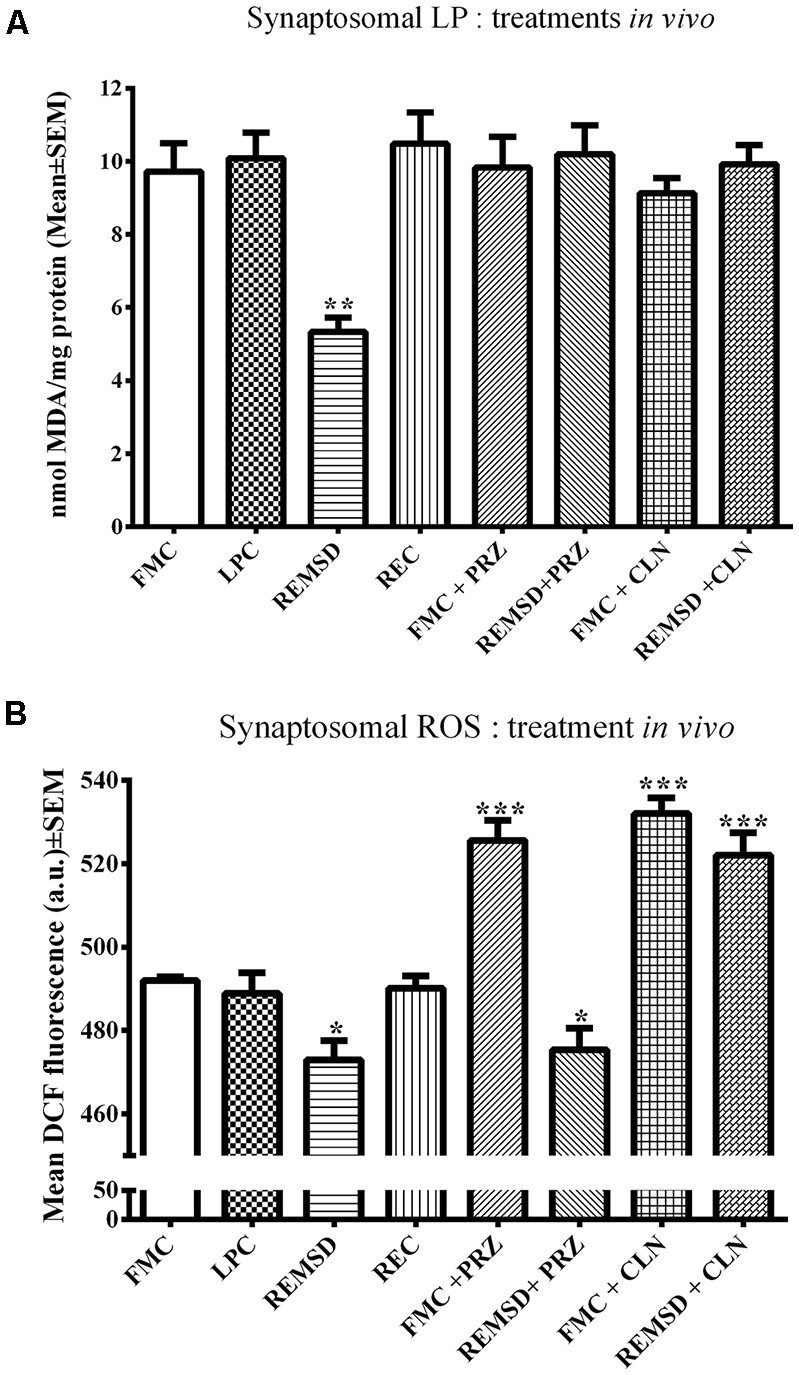
This figure shows relative levels of LP **(A)** and ROS **(B)** in the synaptosomes prepared from the brains of controls and REMSD rats which were treated with or without AR alpha2 agonist (CLN) and alpha1antagonist (PRZ). The treatment details have been described in the method (this was done to reconfirm our earlier findings on set conditions Das et al., 2008). Levels of both LP and ROS were significantly reduced after REMSD. While REMSD associated reduction in LP was prevented by both PRZ and CLN **(A)**, reduction in ROS level was prevented by CLN, but not by PRZ. *N* = 5 for each set. *p < 0.05, **p < 0.01, ***p < 0.001 as compared to FMC. Abbreviations are as in the text.

LP and ROS levels mg^−1^ protein were measured in synaptosomes separately prepared from FMC, LPC, REMSD, and REC rat brains. The group data were significant for both LP (*F*_(3,19)_ = 33.4, *p* < 0.001) and ROS (*F*_(3,19)_ = 5.5, *p* < 0.01). REMSD significantly decreased the levels of LP (*p* < 0.001) and ROS (*p* < 0.01) in synaptosomes as compared to that of the FMC (Figures 1A,B). Both LP as well as ROS levels in synaptosomes prepared from LPC and REC rat brains were comparable to that of the FMC (Figure 1).

#### PRZ and CLN Treatments *in vivo* (i.p.) Prevented REMSD-Induced Reduction in LP and ROS in Rat Brain Synaptosomes

As an elevated level of NA is a primary and common causative factor for many of the REMSD-induced changes (Mallick and Singh, 2011), we investigated its role as well as involvement of AR-subtype in REMSD-induced changes in LP and ROS levels described above. FMC and REMSD rats were intra-peritoneally (i.p.) treated with PRZ or CLN 8 h before sacrifice. The LP levels in these treated groups were comparable to that of the FMC, suggesting that both the treatments prevented the REMSD-induced decrease in LP (Figure 1A).

An intraperitoneal injection of either PRZ or CLN into FMC rats significantly (*F*_(7,32)_ = 29.1, *p* < 0.001) increased ROS levels as compared to untreated FMC rats. The PRZ treatment did not prevent an REMSD-induced reduction in ROS. ROS levels in synaptosomes prepared from CLN-treated REMSD rats were comparable to that of CLN-treated FMC rats (Figure 1B).

### Studies Upon Treatment of Synaptosomes *in vitro*

#### NA Reduced LP and ROS *in vitro* in Synaptosomes Prepared From Normal Rat Brains

**Figure 2:**
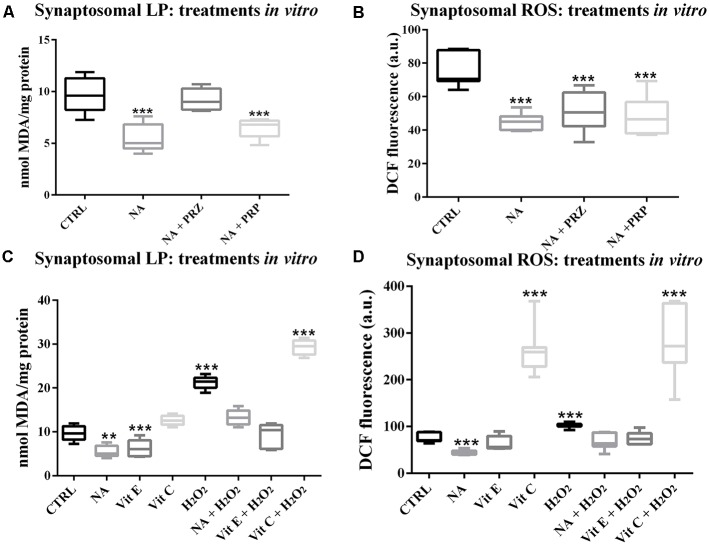
Changes in LP and ROS levels in synaptosomes, which were prepared from FMC rat brains and treated* in vitro* are shown as box-plots. NA (100 μM) significantly decreased both LP and ROS. Although the effect on LP was prevented by PRZ and not by PRP **(A)**, α1- and ß-AR antagonist, respectively, they were ineffective in preventing NA-induce decrease in ROS **(B)**. The effects of NA, Vit E and Vit C on normal and H_2_O_2_-induced changes in synaptosomal LP and ROS have been compared and shown in **(C,D)** respectively. H_2_O_2_ increased LP as well as ROS levels, which were although prevented by pre-treating the synaptosomes with Vit E or NA, the effects were further enhanced if the samples were pre-treated with Vit C. *N* = 5 for each set LP, while *N* = 7 for ROS. **p < 0.01, ***p < 0.001 as compared to control. Abbreviations are as in the text.

To avoid a non-specific effect of PRZ or PRP *in vivo* and to understand the specific involvement of AR, the synaptosomes prepared from FMC rat brains were incubated *in vitro* with either NA, PRZ or PRP and LP as well as ROS levels were compared with untreated synaptosomes taken as controls. As compared to untreated synaptosomes, NA significantly reduced LP (*F*_(1,8)_ = 68.8, *p* < 0.001). Although PRZ (*F*_(1,8)_ = 6.4, *p* < 0.05) prevented the NA-induced decrease in LP, PRP was ineffective (Figure 2A).

Synaptosomes incubated with NA also showed significantly decreased levels of ROS (*F*_(1,8)_ = 85.8, *p* < 0.001). However, the NA-induced decreased ROS levels were not prevented by either PRZ or PRP treatment (Figure 2B).

#### NA and Vit E Reduced ROS in H_2_O_2_-Treated Synaptosomes *in vitro*

We observed in this study (above) that the effect of NA on ROS was not modulated by PRZ or PRP; however, independent reports supported the protective role of NA from the oxidative burden. Therefore, we compared the antioxidative property of NA and other known antioxidants (Vit C and Vit E) on normal as well as on H_2_O_2_-induced LP and ROS *in vitro* in isolated synaptosomes from normal FMC rat brains. As a group, LP was significantly (*F*_(7,32)_ = 99.0, *p* < 0.001) affected. Independently, NA (*p* < 0.05) and Vit E (*p* < 0.05) significantly decreased, while H_2_O_2_ significantly (*p* < 0.001) increased LP in synaptosomes prepared from normal FMC rat brain as compared to the untreated synaptosmes. Further, although NA and Vit E prevented, Vit C significantly (*p* < 0.001) enhanced the effects of H_2_O_2_-induced LP in synaptosomes (Figure 2C).

The ROS levels among control and different treated groups showed significant change (*F*_(7,48)_ = 54.85, *p* < 0.001; Figure 2D). As compared to the untreated (CTRL) synaptosomes, the ROS level significantly decreased upon treatment with NA (*p* < 0.001), while it significantly increased when treated with H_2_O_2_ (*p* < 0.001) or Vit C (*p* < 0.001); Vit E was ineffective. The H_2_O_2_-induced increase in ROS level was prevented by pre-treatment of the synaptosomes with either NA or Vit E; while, Vit C enhanced (*p* < 0.001) the effect of H_2_O_2_ on ROS levels.

### *In vitro* Cell Culture Studies

#### Neuro2a and C6 Cell Viability Was Dependent on NA Doses

**Figure 3:**
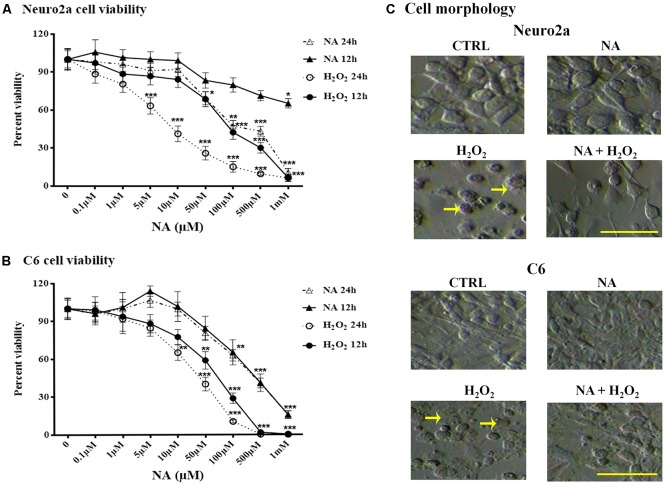
Percentage changes in Neuro2a- and C6-cell viability after 12 h and 24 h treatment with different doses of NA and H_2_O_2_ are shown. Viability of both Neuro2a **(A)** and C6 **(B)** was least affected (<15% of control) by treatment with up to 10 μM of NA, whereas H_2_O_2_ affected the cell viability in even much lesser concentration. *p < 0.05, **p < 0.01, ***p < 0.001 as compared to control. Abbreviations are as in the text. **(C)** Representative microscopic fields of images of Neuro2a and C6 cells with or without treatment with NA (10 μM), H_2_O_2_ or their combination are shown. Both Neuro2a as well as C6 cells showed less adherence to the petri-dish. Those remained adhered, showed membrane granulation and more circular (marked by arrows as representative), which are signs of cellular stress/damage or approaching death upon H_2_O_2_ treatment for 24 h. Cells treated with H_2_O_2_ in presence of NA showed reduced -detachment, -granulation and -circular shaped in addition to lesser death. Scale bar 100 μm.

Dose responses of H_2_O_2_ and NA on Neuro2a and C6 cell viability were tested using an MTT assay. Neuro2a and C6 cells were treated with 0.1–1 mM H_2_O_2_ or NA (doses are shown in the Figures 3A,B) for 12 h and 24 h. The viability of neither Neuro2a (Figure 3A) nor C6 (Figure 3B) cells was affected by low doses (up to 10 μM) of NA; however, it was reduced upon exposure to higher doses (>10 μM) of NA and H_2_O_2_ for 24 h. Morphological features of the cultured cells like surface granulation, relative roundedness and their increased non-adherence (detachment) to the plate surface in the presence of H_2_O_2_ suggested cellular stress and damage, which were prevented if the cells were pre-treated with 10 μM NA (Figure 3C). Based on the dose response findings (Figure 3), we used 10 μM NA for assessing its protective role on H_2_O_2_-induced oxidative damage in Neuro2a and C6 cells.

#### NA (Alpha1 AR Mediated) and Vit E Prevented H_2_O_2_-Induced LP in Neuro2a and C6 Cells

**Figure 4:**
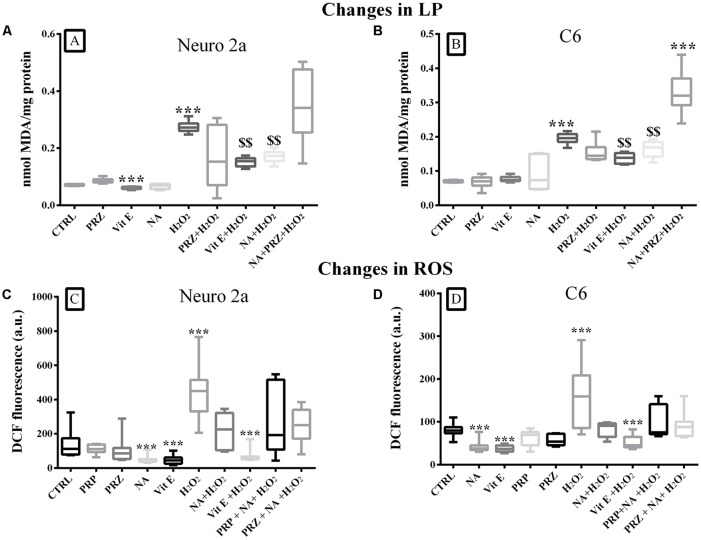
Changes in LP (mean ± SEM nmol of MDA) and intracellular ROS levels (as expressed by DCF fluorescence) in Neuro2a and C6 cells under control and various treatment conditions are shown as box-plots. Confluent cell culture containing about 2 × 10^7^ cells grown in 100 mm petri dishes were treated with Vit E, NA (10 μM), PRZ, H_2_O_2_ alone or in combinations (as shown in respective bars). Vit E (except in Neuro2a), NA and PRZ alone were by and large ineffective in modulating the LP. H_2_O_2_ induced increased LP was reduced by NA and Vit E in both Neuro2a **(A)** and C6 **(B)**. The protective effect of NA on H_2_O_2_ induced LP was lost in presence of PRZ. As shown in **(C,D)** in both Neuro2a and C6 respectively, ROS was increased by H_2_O_2_, while it was decreased by NA and Vit E. The H_2_O_2_ induced effects were prevented by NA and Vit E. As the NA induced effects were not prevented by AR antagonists, PRZ or PRP, it suggested that the effects were not mediated through (ARs). ***p < 0.001 as compared to untreated control and ^$$^*p* < 0.01 in comparison with H_2_O_2_ treated cells. *N* = 4 sets of experiments triplicate readings of ×10^5^ cells for ROS. Abbreviation as in the text.

A group comparison showed that LP was significantly modulated when Neuro2a (*F*_(9,50)_ = 5.3, *p* < 0.001) and C6 (*F*_(9,50)_ = 15.5, *p* < 0.001) cells were treated with Vit E, NA, PRZ, and H_2_O_2_. By and large, H_2_O_2_ significantly (*p* < 0.001) increased, Vit E significantly (*p* < 0.001) decreased (only in Neuro2a), while NA and PRZ were ineffective in modulating LP as compared to untreated controls. Further, NA significantly (*p* < 0.001) reduced H_2_O_2_-induced LP and such a protective effect of NA was lost when the cell lines were pre-treated with PRZ (Figures 4A,B). As PRP was ineffective on LP in synaptosomes, we did not consider it necessary to study its effects on LP on cell lines. This suggested that, by and large, the NA-induced effect on LP was alpha1 AR mediated. Notwithstanding this, on closer look it may be seen that, as an exception, PRZ exaggerated the action of H_2_O_2_ even in the presence of NA (Figure 4B), which needs further investigation to understand the mechanism of action. Thus, Vit E and NA behaved similarly and acted analogously to antioxidant against H_2_O_2_-induced LP in both cell lines. As Vit C was not effective, rather it potentiated the H_2_O_2_-induced damaging effect in synaptosomes, we did not test its effect on cell culture studies.

#### NA (AR Independent) and Vit E Prevented H_2_O_2_-Induced ROS in Neuro2a and C6 Cells

A comparison of intracellular ROS levels in Neuro2a (*F*_(9,70)_ = 7.60, *p* < 0.001) and C6 (*F*_(9,70)_ = 6.43, *p* < 0.001) upon various treatment-groups showed an overall significant difference (Figures 4C,D). In pairwise comparison with respective control cells, H_2_O_2_ significantly increased ROS levels in both, Neuro2a (*p* < 0.001) and C6 (*p* < 0.001) cells, which were prevented if the cells were pre-treated with 10 μM NA or Vit E (Figures 4C,D). Neither PRZ nor PRP (AR-antagonists) prevented the effect of NA. Therefore, as an alternate hypothesis, we investigated the possible involvement of iron on NA-mediated changes in ROS level.

### NA, Vit E, and DFX (Iron Chelator) Prevented H_2_O_2_-Induced ROS in Synaptosomes Prepared From Normal Rat Brain

The ROS levels in synaptosomes, upon various treatments in the presence and absence of DFX, showed a significant (*F*_(7,48)_ = 11.98, *p* < 0.001) difference as a group. A pairwise comparison showed that, as compared to the untreated control, although DFX (Fe^3+^ chelator) decreased synaptosomal ROS levels, it did not reach a statistical significance level (Table 1). However, DFX pre-treatment significantly (*p* < 0.02) reduced H_2_O_2_-induced ROS, which suggests that DFX (Fe^3+^ chelator) prevented the H_2_O_2_-induced generation of ROS. Independently, NA (*p* < 0.001) and Vit E (*p* < 0.001) significantly attenuated the ROS levels in synaptosomes treated with H_2_O_2_ alone. As mentioned above, DFX alone reduced ROS generation, which was further reduced by NA as well as Vit E. Although Vit C potentiated H_2_O_2_-induced increased ROS levels (reported above), it was prevented in the presence of DFX, and the effect was further reduced in the presence of NA (Table 1).

**Table 1:** Modulation of ROS levels in synaptosomes by H_2_O_2_ with or without treatment of NA in presence or absence of Fe^2+^.

Groups/conditions	ROS (Fluorescence a.u.; Mean ± SEM)
CTRL	75.11 ± 3.58
DFX 10 μM	66.46 ± 3.37
H_2_O_2_ 100 μM	102.56 ± 2.04***
DFX 10 μM + H_2_O_2_100 μM	75.68 ± 4.16
DFX 10 μM + NA100 μM + H_2_O_2_ 100 μM	49.10 ± 9.05*
DFX 10 μM + Vit E 100 μM + H_2_O_2_ 100 μM	55.89 ± 4.61**
DFX 10 μM + Vit C 100 μM + H_2_O_2_ 100 μM	65.06 ± 8.39
DFX 10 μM + NA 100 μM + Vit C 100 μM + H_2_O_2_ 100 μM	40.92 ± 4.68***

H_2_O_2_ induced an increase in ROS, while chelation of iron in the presence of DFX blocked the H_2_O_2_ induced ROS generations. Treatment of NA led to additional decrease in ROS levels in DFX treated cells. Either Vit E or Vit C did not affect the H_2_O_2_ induced ROS production. *n* = 7, *p < 0.05, **p <0.01, ***p < 0.001 compared to untreated control (CTRL), abbreviations as in text.

### NA, Vit E and DFX (Iron Chelator) Prevented H_2_O_2_-Induced ROS in Neuro2a and C6 Cells

**Figure 5:**
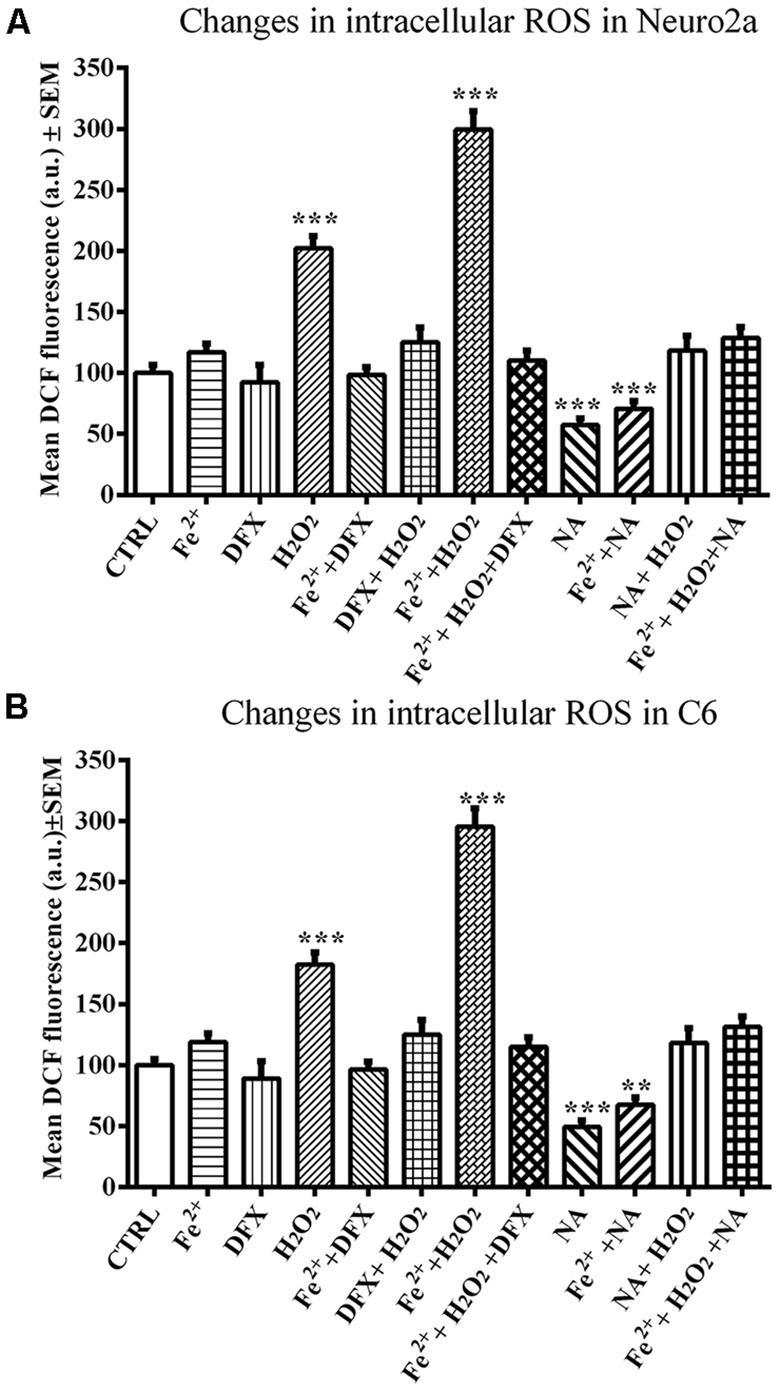
NA mediated modulation of intracellular ROS levels in presence of iron in Neuro2a **(A)** and C6 **(B)** is shown. 2 × 10^5^ cells per well were seeded in 96 well plates and incubated for 12 h in serum free medium lacking iron or in medium containing 10 μM FeSO_4_ as source of iron (Fe^2+^). Cells in both the media were treated with H_2_O_2_ alone or in presence of NA or DFX for 2 h. At the end the cells were incubated with medium containing 25 μM DCFDA for 30 min followed by PBS washing and DCF fluorescence estimation. In the presence of Fe^2+^, H_2_O_2_ enhanced ROS generation in both the cells, Neuro2a **(A)** and C6 **(B)**, which were prevented by DFX and NA. **p < 0.01, ***p < 0.001 as compared with control. Abbreviations are in the text.

A comparison of ROS values as a whole among the groups of control and NA-, DFX- and Fe^2+^-treated Neuro2a (*F*_(11,71)_ = 43.86, *p* < 0.001) and C6 (*F*_(11,71)_ = 42.40, *p* < 0.001) cells showed a significant difference. A pairwise comparison showed that neither DFX nor Fe^2+^ significantly affected ROS in Neuro2a or C6 cells; however, H_2_O_2_ significantly increased ROS levels in both cell lines, Neuro2a (*p* < 0.001), and C6 (*p* < 0.001). NA reduced ROS in both H_2_O_2_-treated as well as normal cells. Further, in Neuro2a and C6 cells, Fe^2+^ (FeSO_4_) and H_2_O_2_ together significantly (*p* < 0.001) increased ROS levels and NA prevented the effects. The prevention of Fe^2+^ and H_2_O_2_-induced ROS generation by NA and DFX was comparable (Figures 5A,B).

### NA inhibited Fe^2+^-Fz Complex Formation

**Figure 6:**
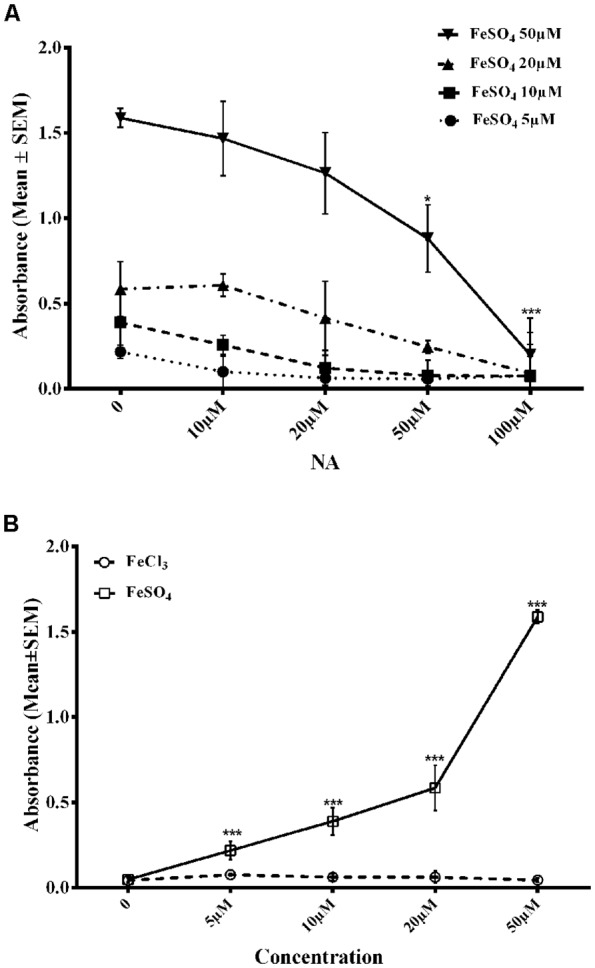
Dose response of NA on reduction in Fe-FZ complex formation is shown. Absorbance of Fe-FZ decreased with addition of increased dose of NA **(A)**. FZ selectively binds to Fe^2+^ and not to Fe^3+^ as shown by increased absorbance in presence of FeSO_4_ and not in the presence of FeCl_3_ **(B)**. The results suggest the Fe^2+^-chelating ability of NA. *p < 0.05, ***p < 0.001 as compared to control. Abbreviations are as in the text.

To confirm the findings reported above, we needed to show if NA could chelate/bind Fe^2+^. FZ is known to selectively chelate Fe^2+^ and the chelated complex can be detected by specific absorbance at 562 nm (Stookey, 1970). On incubating an increasing concentration of FeSO_4_ (5–50 μM) with FZ *in vitro*, there was a dose-dependent increase in absorbance of Fe^2+^-FZ complex (Figure 6). However, when FeSO_4_ was pre-incubated with varying concentrations of NA (10–100 μM) before treating with FZ, there was a dose-dependent decrease in the absorbance as compared to without the treatment of NA (Figure 6A). Still, a higher dose of NA was not tried as the absorbance reached almost zero. To rule out any confounds due to Fe^3+^, we replaced FeSO_4_ with FeCl_3_ (5–50 μM) and treated with FZ, which gave negligible absorbance (Figure 6B). Taken together, all these observations suggested that NA chelated Fe^2+^ that reduced Fe^2+^-FZ complex formation.

### NA Protected Neuro2a and C6 Cell Death by Reducing ROS

**Figure 7:**
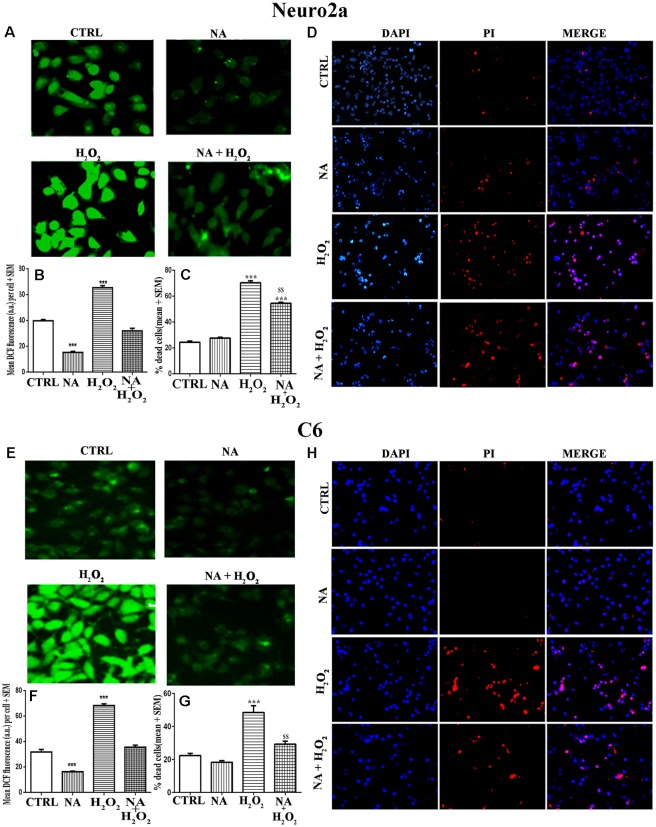
Protective role of NA (10 μM) on H_2_O_2_-induced ROS generation and cell death in Neuro2a (upper panels, **A–D**) and C6 (lower panels, **E–H**) is shown. Representative photomicrographs of intracellular DCF fluorescence indicative of ROS levels in Neuro2a **(A)** and C6 **(E)** are shown. Cells treated with H_2_O_2_ alone showed significantly higher fluorescence per cell, while NA treated cells showed significantly reduced fluorescence per cell upon H_2_O_2_ treatment. Corresponding histograms of relative fluorescence intensities per cell (mean of 500–600 cells per treatment) from five sets of experiments of Neuro2a **(B)** and C6 **(F)** are shown. In separate sets of experiments, the cells were treated for 24 h and percent dead cells were quantified using trypan blue assay. H_2_O_2_ significantly increased cell death, which was protected in presence of NA for Neuro2a **(C)** and C6 **(G)**. Also, using DAPI (stains all live and dead cells) and PI (stains only dead cells) the proportion of dead cells were quantified in coverslip-grown cells treated with NA, H_2_O_2_ or both together. Significantly more number of nuclei were PI (red) stained by treatment of H_2_O_2_ alone as compared to NA alone and NA+H_2_O_2_. Representative photomicrographs (20X) of DAPI and PI stained Neuro2a **(D)** and C6 **(H)** are shown. PI stained cell shown in red are dead while DAPI stained all nuclei irrespective of live or dead are shown in blue; PI stained cells are seen more in H_2_O_2_ treated groups. All readings were taken in duplicate and three (*N* = 3) such sets of experiments were conducted. ***p < 0.001 as compared to untreated cells, while ^$$^*p* < 0.01 as compared to H_2_O_2_ treated cells. Abbreviations are as in the text.

We have seen above that NA reduced the ROS level in Neuro2a and C6. Although reduced ROS is an indicator of prevention or protection from cell death, we confirmed it qualitatively as well as quantitatively using three methods. First, we cultured Neuro2a and C6 cells on coverslips as well as in cell culture plates and treated them for 2 h (for ROS estimation) or 24 h (for evaluation of cell death) with H_2_O_2_ alone or in the presence of NA. Intracellular ROS profile can be seen in photomicrographs (Figures 7A,E). As compared to untreated control cells, H_2_O_2_ significantly (*p* < 0.001) increased ROS levels in both types of cells, which were almost completely prevented by NA (Figures 7B,F). Second, by PI staining qualitatively, it was observed in coverslip-grown cells that NA provided protection to both the cells and prevented H_2_O_2_-induced death (Figures 7D,H). Although PI stained dead cells grown on coverslip could have been quantified, we argued that as the dead cells would tend to detach from the coverslips, the results could be less accurate. To address this issue and to obtain relatively more accurate results particularly in our study we used an additional third method, the trypan-blue assay, to quantify the NA-induced protection of cells. In this method, dead Neuro2a and C6 cells were counted upon treatment with H_2_O_2_ in the presence and absence of 10 μM NA, as described in the “Materials and Methods” section. We observed that as compared to untreated control cells, H_2_O_2_ significantly increased the death of Neuro2a (*p* < 0.001) as well as C6 (*p* < 0.001) cells. Further, as compared with cells treated with H_2_O_2_ alone, the cell death was significantly (*p* < 0.01 for both Neuro2a as well as C6) reduced when they were treated with H_2_O_2_ in the presence of 10 μM NA (Figures 7C,G); the findings confirmed our contention as a proof-of-principle that NA protected the Neuro2a and C6 cells from ROS-induced oxidative damage and death. However, although the cell death by H_2_O_2_ in the presence of NA was significantly lower than treatment with H_2_O_2_ alone, it was higher than the untreated controls, which may appear deceptive. To explain, we argue that complete protection would depend on many contributory factors, including dose and duration of exposure of cells to NA and H_2_O_2_, age and sensitivity of the cells, oxidative inactivation, and the metabolism of NA.

### Dual Response of NA on ROS Generation in C6 Cells

**Figure 8:**
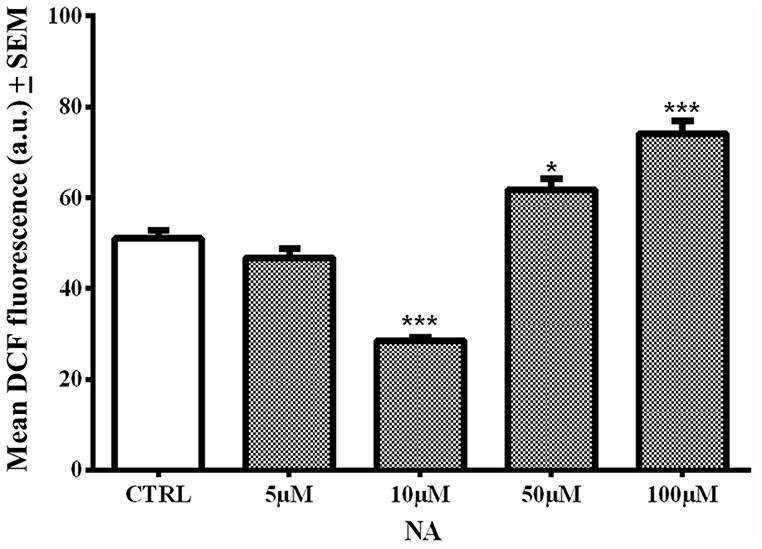
Dose response of NA on ROS generation in C6 cells is shown. At lower concentration (10 μM) NA reduced ROS levels, while at higher concentration (100 μM) there was significant increase in intracellular ROS and it was damaging. *p < 0.05 and ***p < 0.001 in comparison to untreated controls.

In the cell death assay (above), it was observed that 10 μM NA provided incomplete protection of the cells. Therefore, we evaluated the level of ROS generation in C6 cells treated with four doses (5, 10, 50 and 100 μM) of NA alone. We used one of the cells lines (C6 in this case) as a proof-of-principle; synaptosomes were not used as they are non-living. As compared to non-NA treated control cells, the lower doses of NA (5 and 10 μM) were significantly reduced (*p* < 0.001 at 10 μM), while higher doses significantly increased [50 μM (*p* < 0.05) and 100 μM (*p* < 0.001)] intracellular ROS (fluorescence) levels (Figure 8). This indicated that a lower concentration of NA protected the cells, while a higher concentration failed to protect the cells from ROS accumulation, which would be damaging to the cells.

## Discussion

Living tissues routinely carry out metabolic processes, resulting in the continuous generation of ROS as a by-product (Seaver and Imlay, 2004). ROS, being highly reactive, triggers cascading effects, including the induction of LP (Leutner et al., 2001), protein and DNA damage (Valko et al., 2004; Galli et al., 2005), leading to neuronal loss (Casadesus et al., 2004). The vulnerability of the brain to such oxidative damage increases logarithmically because of several reasons including on one hand, the fact that it possesses a large quantity of polyunsaturated fatty acids (the substrate for ROS generation) and it is rich in iron (which favors the Fenton reaction and ROS generation), and on the other hand, it contains significantly less antioxidants to scavenge the continuously generated ROS. It is possible that to compensate at least partially, the brain has been gifted with a natural protector, the NA, possessing antioxidant property (Troadec et al., 2001; Traver et al., 2005). Therefore, we investigated *in vivo* and *in vitro* NA-ergic modulation of LP and ROS levels with and without the involvement of ARs and the role of iron. Also, we investigated the effect of NA because its level changes with a unique physiological process regulated by the brain, the REMS and its loss (Mallick et al., 2002, 2012). The results of our *in vitro* studies including those on cell-lines, complement the findings from *in vivo* animal studies. They suggest that low level of NA protects the brain (neurons and glia) from oxidative insult by reducing LP and ROS, while a high level of NA-induced cell death.

In this study, we observed that both LP and ROS decreased in synaptosomes prepared from REMS-deprived rat brains. PRZ, by binding on the post-synaptic alpha1-AR, prevented the released NA to induce its action (Greengrass and Bremner, 1979), while CLN, by acting on the pre-synaptic alpha2-AR, inhibited the release of NA (Washburn and Moises, 1989; Tomasini et al., 1992; Wang et al., 2017); thus, the resultant effect of both PRZ and CLN was similar although the mechanism of action differed. A closer evaluation of the results, particularly with PRZ and CLN, suggest that the NA-induced effect on LP was AR-mediated, while the effect on ROS was both AR and non-AR mediated; the latter effect could be due to the antioxidant property of NA. To understand the complex mechanism of action, as a first step, in the cell-culture model we used H_2_O_2_ to induce ROS and LP within live cells. The H_2_O_2_ was chosen as it is membrane permeable, endogenously produced as well as metabolized, it is known to induce ROS and its associated cellular consequences (Antunes and Cadenas, 2000; Ullrich and Kissner, 2006). The H_2_O_2_-induced enhanced ROS and LP productions were prevented by NA, which supported the antioxidant property of the latter.

Vit E, Vit C and glutathione scavenge free radicals and ROS to protect tissues from oxidative damage. We compared the effects of NA, Vit E, and Vit C on H_2_O_2_-induced ROS generation. Vit E is a potent antioxidant (Koul et al., 2005; Singh et al., 2005), while Vit C is reported to behave as pro- as well as anti-oxidant (Halliwell, 1996; Carr and Frei, 1999). We observed that in the absence of H_2_O_2_, Vit E decreased ROS levels in synaptosomes similar to that of NA; while Vit C induced opposite effects. Further, both NA and Vit E individually as well as together prevented the H_2_O_2_-induced increase in ROS levels, while Vit C potentiated the H_2_O_2_ effects. These findings, however, suggest that NA apparently acts analogously to that of Vit E to decrease ROS, the precise mechanism of action needed elaborate study. It is generally known that NA as neurotransmitter usually acts through ARs. Although non-AR mediated action of NA has been proposed infrequently, its mechanism of action particularly *in vivo* and its relation with REMS were unknown, which has been confirmed in this study.

The brain is rich in the essential nutrient, iron, which facilitates generation of ROS through *Fenton* reaction (Haber and Weiss, 1932); therefore, an optimum level of iron is necessary for maintaining normal physiological processes. An elevated iron is associated with neuro-degenerative diseases (Castellani et al., 2004; Zecca et al., 2004), while iron deficiency including anemia is associated with restless leg syndrome (Lee et al., 2001; Allen and Earley, 2007; Patrick, 2007; Auerbach, 2014; Connor et al., 2017); interestingly, in all those diseases, REMS is adversely affected. As REMS maintains brain NA level (Mallick and Singh, 2011; Mehta et al., 2017) and the latter possesses catechol ring that can chelate iron, we explored the relationship between NA and iron levels in modulating ROS in the brain, importantly an antioxidant compromised organ.

As iron exists in the body in Fe^3+^ and Fe^2+^ forms, we explored their specificity in providing protection to the brain from oxidative insult (as evidenced by ROS generation). Either DFX, a specific Fe^3+^ chelator or NA and their combination not only reduced ROS level, they also prevented the potentiation of ROS formation by H_2_O_2_ in synaptosomes. Subsequently, we confirmed the findings on Neuro2a and C6 (living) cells, which have been routinely used as models for understanding neuronal- and glial-cellular physiology, respectively. We observed that H_2_O_2_-induced LP and ROS levels were prevented by NA and Vit E. Further, as the protective effect of NA was comparable in synaptosomes and in cultured (living) cells, it is expected that a similar mechanism operates in the brain *in vivo* as well; however, this needs to be confirmed. Thereafter, we embarked on understanding the involvement of Fe^2+^ and the mechanism of action of NA in providing the protection. We demonstrated that the Fe^2+^ enhanced H_2_O_2_-induced ROS generation in both, Neuro2a and C6 cells and the effect was prevented by NA and DFX, possibly by the chelation of iron (Fe^2+^/Fe^3+^) in the medium, as classically the latter is known to chelate Fe^3+^.

By estimating the Fe-FZ complex, we confirmed that NA chelated Fe^2+^ (not Fe^3+^) and prevented the *Fenton* reaction from inducing ROS even in the presence of H_2_O_2_ and thus, the oxidative load of the cells was reduced. This finding may be supported by an earlier report that amines including NA possesses a divalent ion-chelating property (García et al., 2012). It may be argued that isolated, scattered report (Andersson et al., 1988) has shown possible indirect interaction between Fe^3+^ and NA under a certain specific condition and that might influence the findings. However, in our experimental condition such interaction is an unlikely possibility and may need to be confirmed. Finally, to confirm if our proposed mechanism really works to prevent cell-death, we treated cultured Neuro2a and C6 cells with H_2_O_2_ in the presence and absence of NA and quantified dead/live cells. We observed that the presence of NA indeed reduced ROS as well as cell-death induced by H_2_O_2_ alone (Figure 7). Although this observation supported our contention as a proof-of-principle that NA protected the cells from the oxidative burden and cell death, one may argue that the protection was incomplete. We argue that as a living system is exposed to multiple opposing metabolic modulators, total protection could possibly be obtained by varying one or more factors e.g., the dose, duration of exposure and oxidation level of NA, the metabolic state, sensitivity, age and predisposition of the cells and other non-specific factor(s) in the cellular milieu, which was not the primary aim of this study and needs to be investigated.

Therefore, we further evaluated the dose response of NA on normal ROS generation in C6 cells. A lower dose (≃10 μM) of NA prevented, while a higher dose (≃100 μM) failed to prevent the elevation of ROS as compared to normal conditions in the C6 cells. The dual response of NA on ROS generation was dependent on the concentration of the compound and may be supported by C6 viability (Figure 3B). Further, as a critical evaluation we should mention that in our study with synaptosomes (Figures 2, 3 and Table 1), 100 μM of NA (the dose was selected to start with based on our previous report with synaptosomes) reduced ROS generation in synaptosomes, which may deceptively appear to be non-supportive. The reasons could be that: (i) earlier we did not carry out dose response of NA on synaptosomes; and (ii) as compared to living cells, the synaptosomes differ in their -milieu, -metabolism, -oxidation/ reduction levels and -dynamicity. For living cells, although the precise protective concentration of NA may not be readily predicted, our findings support, in principle, that at least a lower dose of NA reduces the ROS level and provides protection to the brain, while a higher dose including upon REMSD, is destructive; the latter even *in vivo* has been reported to act through alpha-ARs (Biswas et al., 2006; Ranjan et al., 2010; Somarajan et al., 2016).

We used the flower-pot method, the most practical and globally preferred method of choice for long-term REMSD with least human intervention. The arguments offered in favor of the observed effects being specific to REMSD (Vogel, 1975; Gulyani et al., 2000; Mehta et al., 2018) hold true for this study also. Further, the observed effects were due to REMSD because the changes returned to normal level in the REC rats. To rule out unavoidable, undesirable, confounding non-specific effects including some loss of non-REMS and stress, if at all, we carried out LPC group (control). The LPC and REMSD rats suffer comparable non-specific influence and the former suffer a non-significant loss of REMS, particularly when maintained on the large platform for longer than 48 h (Mendelson et al., 1974). As LP and ROS in LPC and FMC rats were comparable, while the effects in experimental rats were significantly different, the effects of non-specific factors on REMSD rats can be safely ruled out. Notwithstanding this, a few isolated conflicting findings have been reported e.g., one study showed no change (D’Almeida et al., 1997), while another showed a higher level (Mathangi et al., 2012) of LP upon REMSD. The differences in results are likely due to non-compliance with animal-weight to the platform-size ratio as has already been reported (Mehta et al., 2018). A specific ratio of body weight to platform size is of critical importance for inducing REMSD to the experimental animals and for the LPC animals to serve as an ideal control (Yanik and Radulovacki, 1987); hence, the results of the two studies cannot be compared. Besides, our contention gets support by another report in which as the desired body weight to platform size ratio was maintained even for higher weight group of rats, the animals expressed reduced LP (Singh et al., 2008).

To avoid perceived possible stress, in isolated studies (Khadrawy et al., 2011; Suer et al., 2011; Zhang et al., 2013), multiple platforms were used for REMSD (unlike the single platform used in most, including this, study); however, the results were inconsistent. The differences in results could be due to the fact that the experimental protocol in those studies suffered various issues including body weight to platform size ratio, lack of adequate controls, lack of recovery group, prevention of the induced change by AR-antagonist, which have been carefully taken care of in this study to rule out non-specific effects. Also, it may be noted that unlike use of single platform used in this and our earlier studies (Das et al., 2008), where there was reduced LP, in most of the studies where multiple platforms were used (Khadrawy et al., 2011; Suer et al., 2011; Zhang et al., 2013), there have been increased LP, which needs to be carefully interpreted and investigated. One obvious reason for the difference in results is that as the animal body weight to platform size was not maintained, the LPC rats were also REMSD, thus adequate control was missing. Additionally, our yet unpublished observation that in a multiple platform method, because of the presence of more than one animal in the same enclosure, one animal jumps to anther platform where other animal rests/sleeps and thus, animals on small as well as large platforms are sleep deprived, support our contention. Finally, electrophysiological sleep-waking recordings of rats on flower-pot (as used in this study) confirmed selective REMS loss of the experimental rats and importantly, the effects were not due to stress (Porkka-Heiskanen et al., 1995; McDermott et al., 2003; Kitka et al., 2009). This supports our contention that the effects observed in this study were due to the REMSD of the rats.

## Summary

**Figure 9:**
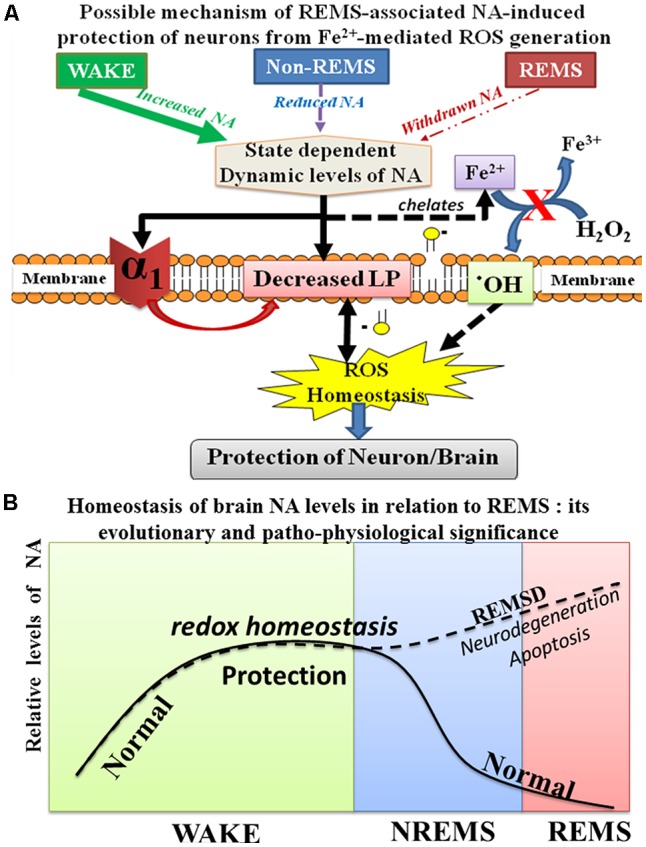
Schematic illustration shows the possible relationship of NA-mediated neuroprotective mechanism during sleep and waking. During waking as the brain is metabolically more active, more ROS is generated; however, continuous release of NA from the NA-ergic REM-OFF neurons protects the brain from oxidative damage. NA prevents ROS generation in cells by reducing α1-AR mediated reduction in LP as well as by possible chelation of Fe^2+^ **(A)**. Further during REMS, NA-ergic REM-OFF neurons slow-down and stop activity resulting in washing-off the accumulated NA to maintain homeostasis and the cycle repeats **(B)**. Thus, the finding helps explaining role of REMS in maintaining brain NA level. Abbreviations are as in the text.

The brain is metabolically more active than other organs and being an antioxidant compromised organ, it is more sensitive as well as vulnerable to oxidative load. It has been reported earlier that REMSD-induced elevated NA caused apoptosis in rat brains (Biswas et al., 2006; Ranjan et al., 2010; Somarajan et al., 2016) and induced neuro-degenerative disorders (Postuma et al., 2013; Pillai and Leverenz, 2017); however, the molecular mechanism of action was unknown. On the contrary, NA is known to possess antioxidant property. Therefore, we explored the dose-dependent possible mechanism of action of NA in preventing an oxidative load induced damage to neurons and glia. As shown in Figure 1 (*in vivo* study), REMSD decreased ROS (and LP), which was prevented by CLN (which reduces NA release) and PRZ (which prevented NA to act on alpha1 AR). We then went on to decipher the possible mechanism of action of NA (Figure 9A), which we carried out *in vitro* on synaptosomes and cell lines for obvious reason that all variables cannot be controlled *in vivo*. The findings of this study that a lower dose of NA prevents conversion of Fe^3+^ to Fe^2+^ and reduces ROS generation through *Fenton* reaction and prevents neuronal death partly help in resolving the paradox. However, further *in vivo* studies are needed to confirm our *in vitro* results and to understand why and how a higher dose of NA induces more ROS.

## Physiological Significance and Conclusion

The findings of this study, together with our knowledge of neural regulation and function(s) of REMS (Mallick and Singh, 2011; Mallick et al., 2012), help us to conclude as follows. During the day when the brain is more active, a continuous release of NA from REM-OFF neurons inhibits the *Fenton* reaction to maintain the generation of ROS under control and thus, the effect of oxidative onslaught on the brain is reduced. As continuous activity of those NA-ergic neurons through the day tends to elevate the NA level, which would damage the neurons and glia (mechanism yet to be deciphered), the REM-OFF neurons cease activity to wash off or at least significantly reduce the level of NA in the brain and this stage is REMS. Thus, essentially, REMS maintains the NA level possibly to protect the brain. As the critical level of NA (set-point) is in dynamic equilibrium with many associated patho-physiological processes and cellular recovery, we refrain from commenting on the said set-point. Our findings are proof-of-principle that provide the basic scaffold to explain, at least partly, the molecular mechanism of REMS-associated protection of the brain, particularly involving NA (Figure 9B).

## Data Availability

All datasets generated for this study are included in the manuscript.

## Author Contributions

BM conceived the problem and arranged funds for the study. AS, GD and MK carried out the experiments and analyzed the data. All authors contributed to preparing the manuscript, read and agreed with its content.

## Conflict of Interest Statement

The authors declare that the research was conducted in the absence of any commercial or financial relationships that could be construed as a potential conflict of interest.

## Footnotes

^1^http://www.ncbi.nlm.nih.gov/books/NBK54050/ doi: 10.17226/12910

## Abbreviations

AR: adrenoceptor
BHT: butylated hydroxyl-toluene
CLN: clonidine
DAPI: 6-diamidino-2-phenylindole dihydrochloride
DCF: dichlorofluorescein
DCF-DA: 2′,7′-dichlorofluorescein diacetate
DCFH: reduced dichlorofluorescein
DFX: desferrioxamine
DMEM: Dulbecco modified Eagle’s medium
DMSO: Dimethyl sulfoxide
FBS: fetal bovine serum
FeCl_3_: ferric chloride
FeSO_4_: ferrous sulfate
FMC: free moving control
FZ: ferrozine
H_2_O_2_: hydrogen peroxide
LP: lipid peroxidation
LPC: large platform control
MDA: malonyldehyde
MTT: 3-(4,5-dimethylthiazol-2-yl)-2,5-diphenyltetrazolium bromide
NA: noradrenaline
PBS: phosphate buffer saline
PI: propidium iodide
PRP: propranolol
PRZ: prazosin
REC: recovery control
REMS: rapid eye movement sleep
REMSD: REMS deprivation
ROS: reactive oxygen species
TBA: thiobarbituric acid
TCA: trichloracetic acid
Vit C: vitamin C
Vit E: vitamin E.
